# Chromosome-Level Genome Assembly of Eden's Whale Clarifies the Taxonomy and Speciation of Bryde's Whale Complex

**DOI:** 10.1093/molbev/msaf234

**Published:** 2025-09-18

**Authors:** Yi-Tao Lin, Fan Hui, Wentao Han, Yi-Xuan Li, Bonnie Yuen Wai Heung, Chun Ming How, Shi Wang, Jian-Wen Qiu

**Affiliations:** Department of Biology, Hong Kong Baptist University, Hong Kong SAR 999077, China; Department of Biology, Hong Kong Baptist University, Hong Kong SAR 999077, China; Southern Marine Science and Engineering Guangdong Laboratory (Guangzhou), Guangzhou 511458, China; Fang Zongxi Center for Marine Evo-Devo & MOE Key Laboratory of Marine Genetics and Breeding, Ocean University of China, Qingdao 266100, China; Laboratory for Marine Biology and Biotechnology, Qingdao Marine Science and Technology Center, Laoshan Laboratory, Qingdao 266100, China; Department of Biology, Hong Kong Baptist University, Hong Kong SAR 999077, China; Department of Biology, Hong Kong Baptist University, Hong Kong SAR 999077, China; Department of Biology, Hong Kong Baptist University, Hong Kong SAR 999077, China; Southern Marine Science and Engineering Guangdong Laboratory (Guangzhou), Guangzhou 511458, China; Fang Zongxi Center for Marine Evo-Devo & MOE Key Laboratory of Marine Genetics and Breeding, Ocean University of China, Qingdao 266100, China; Laboratory for Marine Biology and Biotechnology, Qingdao Marine Science and Technology Center, Laoshan Laboratory, Qingdao 266100, China; Department of Biology, Hong Kong Baptist University, Hong Kong SAR 999077, China

**Keywords:** *Balaenoptera*, cetacean, evolution, marine mammal, Mysticeti

## Abstract

Eden's whale (*Balaenoptera edeni*), a poorly understood baleen cetacean, has long been shrouded in taxonomic ambiguity due to limited genomic resources, obscuring its distinction from closely related species and its position within the cetacean Tree of Life. In this paper, we present a high-quality chromosomal-level genome of *B. edeni* and conduct comparative genomic analyses to address long-standing taxonomic confusion and elucidate speciation of balaenopterids. Our phylogenomic analysis and demographic reconstruction reveal that *B. edeni* is a distinct sister to Bryde's whale (*Balaenoptera brydei*), sharing a common ancestor that diverged approximately 7.84 million years ago during the late Miocene. Their genetic divergence exceeds typical intraspecific variation in whales, supporting the reinstatement of *B. brydei* as a valid species. Chromosomal syntenic analyses suggest that macro-fragment inversions contributed to speciation in balaenopterid whales and uncover unexpected large-scale complex genome rearrangements in Bryde's whale, offering novel insights into cetacean genome evolution. Functional enrichment analysis of inverted regions between *B. edeni* and *Balaenoptera musculus* indicates their predominant association with metabolism and biosynthesis, as well as responses to various substances, stress, and stimuli. These genomic resources for *B. edeni* not only lay a critical foundation for comparative genetic and evolutionary research of cetaceans but also advance our understanding of the taxonomy and evolutionary dynamics of the Bryde's whale complex, with broader implications for baleen whale conservation and biodiversity.

## Introduction

Cetaceans, which evolved from terrestrial ancestors, are divided into baleen and toothed whales (Mysticeti and Odontoceti, respectively), with their varied sizes and distributions reflecting adaptations to diverse marine environments (Marx and Fordyce 2015; Park et al. 2015; Perrin et al. 2018; Davis 2019; Wolf et al. 2022). Within the genus *Balaenoptera*, the taxonomy of the Bryde's whale complex has attracted significant attention over the past decades (Sasaki et al. 2006). Initially, Anderson (1878) described *Balaenoptera edeni* based on a mature specimen measuring 11.3 m, while *Balaenoptera brydei* was described based on individuals with sizes from 12.4 to 15.0 m, larger than *B. edeni* at sexual and physical maturity (Olsen 1913; Best 1977). Subsequent studies suggested that *B. edeni* and *B. brydei* were subspecies of the same species based on skeletal structures, ie *Balaenoptera edeni edeni* and *Balaenoptera edeni brydei* (Andrews 1918; Junge 1950; Kato and Perrin 2009; Kershaw et al. 2013). In this view, the smaller *B. e. edeni* was thought to inhabit the coasts of the Western Pacific and Indian Oceans, while *B. e. brydei* was considered the larger, more widely distributed form (Sasaki et al. 2006; Kato and Perrin 2009; Kershaw et al. 2013). Conversely, other morphological studies argued that Eden's and Bryde's whales should be considered distinct species due to differences in cranial structures (Soot-Ryen 1961; Wada et al. 2003; Rosel and Wilcox 2014; Rosel et al. 2021). Furthermore, the discovery of two additional species within the Bryde's whale complex, *Balaenoptera omurai* from the Solomon Islands and the Eastern Indian Ocean, and *Balaenoptera ricei* from the Gulf of Mexico, further complicated the taxonomy of this group (Wada et al. 2003; Rosel et al. 2021). Phylogenetic studies utilizing mitogenomic fragments have provided further evidence for the distinctiveness of Eden's and Bryde's whales, suggesting that *B. edeni* is sister to *B. ricei* instead of *B. brydei*, while *B. omurai* is the earliest diverging lineage within the Bryde's whale complex (Wada et al. 2003; Sasaki et al. 2006; Luksenburg et al. 2015; Rosel et al. 2021). Despite these findings, *B. brydei* is not yet formally recognized by the Committee on Taxonomy from the Society for Marine Mammalogy (https://marinemammalscience.org/) and is currently categorized as a synonym of *B. edeni* in WoRMs (https://www.marinespecies.org) due to limited genetic data (Wada et al. 2003; Rosel et al. 2021).

In contrast to other balaenopterid species like *Balaenoptera acutorostrata* and *B. musculus*, which have been extensively analyzed through genomic studies (Park et al. 2015; Attard et al. 2018; Bisconti and Carnevale 2022; Bukhman et al. 2024; Jossey et al. 2024; Wolf et al. 2025), the genomics and molecular biology of Eden's whale remain poorly explored. Only a few studies have focused on its distribution, behavior, and genetics (Ren et al. 2022; Chen et al. 2023; Sun et al. 2023; Ibrahim et al. 2024). Notably, Eden's whale is among the few species within the genus *Balaenoptera* that lack an assembled genome. By utilizing tissues from an Eden's whale carcass, we sequenced and assembled its genome at the chromosomal level to study the taxonomy and speciation of the family Balaenopteridae. These genomic resources enable us to examine the chromosome structure of Eden's whale and compare it with other species in the Bryde's whale complex, providing substantial evidence for the reinstatement of *B. brydei* as a distinct species. Accurate species delimitation will facilitate better determination of their distribution ranges, population sizes, and conservation units.

## Results

### High-Quality Genome Assembly and Genetic Distance

**Fig. 1.**
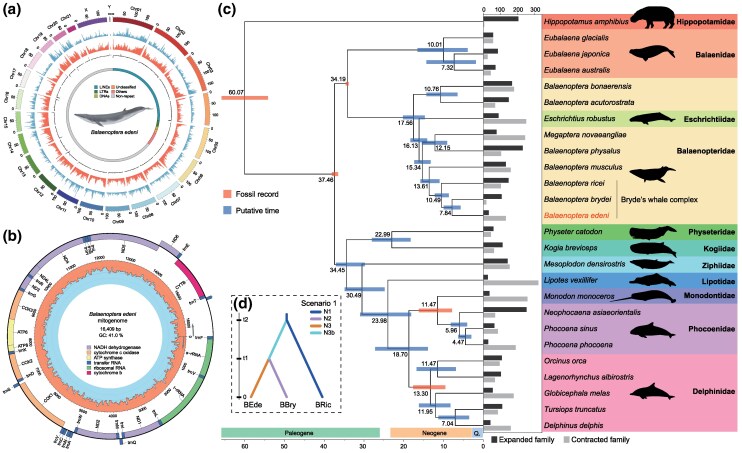
Genomic structure and phylogenomic relationships of Eden's whale. a) Circus plot of 23 linkage groups (corresponding to chromosomes) showing marker distributions at 1 Mb sliding windows from outer to inner circle: gene density, GC content, and GC skew. The animal icon in the center of the circus plot was obtained from the Plazi's databases (https://plazi.org/) without copyright restriction. b) Structure of the mitogenome showing protein-coding genes and RNAs. c) Phylogenomic relationships, divergent times, and expanded and contracted gene families of cetaceans with hippo as the outgroup. The tree was constructed using 3,360 single-copy orthogroups. The bootstrap values are 100 at all nodes. The tree was fossil-calibrated at five nodes. The bar length indicated the 95% confidence interval. Sources and characteristics of the genomes are included in Table S2. The bar plot shows the number of significantly expanded and contracted gene families in each species relative to its nearest node, with a *P*-value < 0.05. The animal icons do not represent their real body sizes. They were obtained from the Phylopic website (https://www.phylopic.org/) without copyright restriction. d) The best putative divergence scenario of Bryde's whale complex (Scenario 1) based on the randomly sampled SNP dataset (*N* = 10,295). Other scenarios are shown in Fig. S3. N1, *Balaenoptera ricei* (BRic); N2, *B. brydei* (BBry); N3, *B. edeni* (BEde); N3b, putative mimics demographic bottleneck; t2, time point of first divergence event from the ancestor; from t2 to 0, from the ancient to present.

Our assembly produced a chromosomal-level genome comprising 23 chromosomes (21 autosomes and allosomes X and Y, 2*n* = 44), with a size of 2.2 Gb (Fig. 1a and Fig. S1, Table 1 and Tables S1 to S3). Gene prediction identified 19,476 protein-coding genes (Fig. S1, Table 1). Benchmarking Universal Single-Copy Orthologs (BUSCO) assessment confirmed the high quality of the assembly (99.70% complete BUSCOs) and predicted gene models (97.50% complete BUSCOs). Additionally, we assembled the mitogenome of *B. edeni*, which is 16,409 bp in length and contains 13 protein-coding genes (Fig. 1b and Fig. S1). This mitogenome exhibits 99.98% identity with the previously published *B. edeni* mitogenome (GenBank accession: AB201258), further validating the taxonomic accuracy of our assembly. We estimated the Kimura 2-parameter (K2P) genetic distances using available complete *cox1* sequences of cetaceans to elucidate interspecific divergence (Table S4). Our results revealed that the K2P genetic distance is 0 among the three specimens of *B. edeni* available for comparison and varies between 0% and 0.13% among the three specimens of *B. brydei*. In contrast, the *cox1* genetic distance between these two species reaches 2.94% (Table S4). The *cox1* K2P genetic distance between *B. edeni* and *B. ricei* is 1.81%, in stark contrast to the three *Eubalaena* whales (*E. japonica*, *E. australis*, and *E. glacialis*), which exhibit minimal interspecific distances (0.52% to 0.86%), notably lower than the divergence observed between Eden's and Bryde's whales.

**Table 1.** Genome assembly and annotation statistics

Item	Number
**Contig assembly**	
Total length (Mb)	2,616.0
No. of contigs	163
GC content (%)	41.3
N50 (Mb)	60.8
Max. length (Mb)	154.1
Min. length (kb)	29.9
Average length (Mb)	16.0
**Chromosome assembly**	
Total length (Mb)	2,614.8
No. of Chromosome	23
No. of Scaffold	105
GC content (%)	41.3
N50 (Mb)	112.3
Max. length (Mb)	188.9
Min. length (kb)	25.0
Average length (Mb)	20.4
Genome coverage	43.7×
Mapping rate	99.98%
BUSCO cetartiodactyla_odb10	C: 99.7%; F: 0.1%; M: 0.2%
**Genome annotation**	
Protein-coding genes	19,476
Average gene length	1,618
With annotation	19,155 (98.35%)
BUSCO cetartiodactyla_odb10	C: 97.5%; F: 0.3%; M: 2.2%

### Fossil-Calibrated Phylogenomic Tree and Demographic Analyses Reveal Species Divergence History

Phylogenomic reconstruction based on 3,360 single-copy orthogroups across 25 cetaceans, along with *Hippopotamus amphibius* as the outgroup, revealed a topology consistent with previous phylogenomic studies of cetaceans, supported by high bootstrap values (100 for all nodes) (Fig. 1c and Fig. S1). Within Balaenopteridae, *B. edeni* is phylogenetically close to *B. brydei*, forming a clade sister to Rice's whale (*B. ricei*). Notably, the balaenopterid humpback whale *Megaptera novaeangliae* and the eschrichtiid gray whale *Eschrichtius robustus* are nested within the *Balaenoptera* clade (Fig. 1c). The clade comprising Balaenopteridae and Eschrichtiidae is sister to Balaenidae, together forming the Mysticeti clade. Calibration of the tree using fossil records indicated that the common ancestor of *B. edeni* and *B. brydei* diverged approximately 7.84 million years ago (Ma) (95% confidence interval = 5.67 to 9.94 Ma) during the late Miocene, whereas the split between their ancestral lineage and *B. ricei* occurred earlier, around 10.49 Ma. The species historically considered part of the Bryde's whale complex, including *B. edeni*, *B. brydei*, and *B. ricei* (genome of *B. omurai* is unavailable), diverged earlier than the divergence between harbor porpoise *Phocoena phocoena* and vaquita *Phocoena sinus* (4.47 Ma), similar to the divergence of balaenid whales *Eubalaena* spp. (Fig. 1c). Our results support *B. edeni* and *B. brydei* as two distinct species, with a genetic divergence comparable to or even higher than that between other congeneric sister species.

To elucidate the evolutionary history of Bryde's whale complex, we determined the most likely demographic scenario among *B ricei*, *B. brydei*, and *B. edeni* using genome-wide SNP data. Among six examined scenarios (Figs. S3 and S4), Scenario 1, characterized by successive divergences without subsequent population bottlenecks, received the strongest support (posterior probability = 0.9956, Fig. 1d). In this scenario, *B. brydei* and *B. edeni* shared a most common ancestor at t1 (the most recent divergence time), and this common ancestor is sister to *B. ricei*, which diverged from their most recent common ancestor at t2 (the most ancient divergence time among the three species). This scenario aligns with the phylogenetic topology (Fig. 1c) and suggests that the divergence history of Bryde's whale complex is well explained by clean and sequential splits without additional demographic complexity.

### Chromosomal Structure Variation among Balaenopterids

**Fig. 2.**
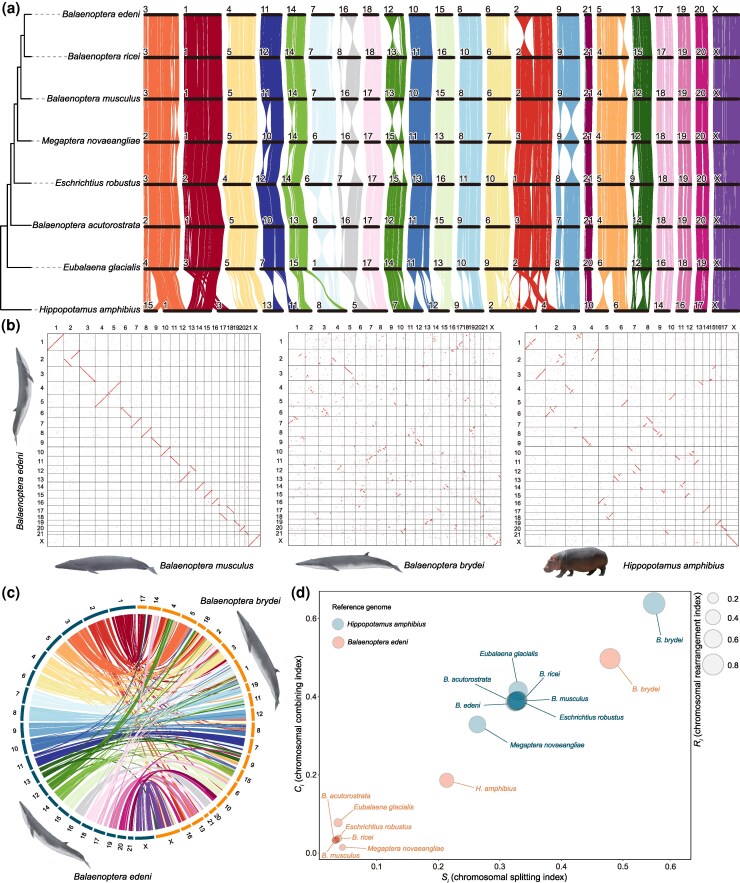
Synteny relationship between Eden's whale and its relatives. a) Ribbon diagram showing the microsynteny between the chromosomes of Eden's whale and its relatives. b) Macrosynteny between Eden's, blue, and Bryde's whales, and the hippo. c) Inter-chromosomal synteny between Eden's and Bryde's whales. d) Chromosomal rearrangement index of selected genomes references Eden's whale and hippo, respectively. The animal icons were obtained from the Plazi's databases (https://plazi.org/) without copyright restriction.

Chromosomal synteny provides valuable insights into genomic evolution. Compared to the outgroup *H. amphibius*, which possesses 18 chromosomes (2*n* = 36), the genomes of the Mysticeti clade exhibit diverse structural variations, including breakages, insertions, inversions, and fusions (Fig. 2a). Despite these variations, the chromosome number in mysticetes remains largely conserved, with exceptions noted for fusions in the genomes of *Eubalaena* spp. (Fig. 1a to c, Table S2). All examined balaenopterid and eschrichtiid genomes consist of 21 pairs of autosomes and one pair of allosomes (2*n* = 44). Our microsynteny analysis of balaenopterid genomes revealed that most chromosomal structures are highly conserved within the family, particularly Chr1, Chr8, Chr10, Chr15, Chr17, Chr18, Chr19, Chr20, Chr21, and ChrX based on the chromosome numbering of *B. edeni* (Fig. 2a). However, several macro-fragment inversions were identified across species. Notably, each balaenopterid genome exhibits at least one inter-chromosomal macro-fragment inversion compared to its closest relatives. Specifically: (i) Chr8 of the common minke whale (*B. acutorostrata*) contains a significantly inverted region compared to other balaenopterid whales; (ii) Chr9, Chr10, and Chr14 of *M. novaeangliae* exhibit inversions relative to other balaenopterid species; (iii) in *B. musculus*, a small region of Chr3 is inverted compared to *B. acutorostrata*, while the inversions in Chr4, Chr7, and Chr8 compared to *B. ricei*, and Chr2, Chr5, Chr6, Chr13, and Chr16 compared to *B. edeni*, distinguish it from Eden's and Rice's whales; (iv) in Rice's whales, Chr2, Chr4, Chr8, and Chr13 show inversions compared to *B. edeni*. Unexpectedly, we found large-scale complex rearrangements in the genome of Bryde's whale (Fig. 2b and c), although we cannot rule out the possibility that these are artifacts arising from its genome misassembly. Notably, intra-chromosomal inversions and inter-chromosomal insertions frequently occur across Bryde's whale chromosomes compared to *B. edeni*, leading to a complex and nearly disordered syntenic relationship between Bryde's whale, other cetaceans, and the outgroup hippo (Fig. 2). In *B. brydei*, only chromosomes Chr4, Chr5, Chr10, Chr12, Chr20, and Chr21 maintain large syntenic blocks aligned with *B. edeni*, with Chr5 containing the largest syntenic block in the whole genome (Fig. 2b and c). Additionally, we used the rearrangement index (*R_i_*) to quantify and compare the rearrangement levels among selected cetaceans. Analyses using different reference genomes, *B. edeni* and *H. amphibius*, yielded similar results. Notably, *B. brydei* exhibits a significantly higher chromosomal rearrangement level than other Mysticeti species, as evidenced by its largest *R_i_*, *C_i_*, and *S_i_* values (Fig. 2d). Moreover, in *B. brydei* and *E. glacialis*, the *C_i_* values are larger than the *S_i_* values, indicating a predominance of intra-chromosomal rearrangements. In contrast, in *M. novaeangliae*, the *C_i_* values are smaller than the *S_i_* values, indicating frequent inter-chromosomal inversions. The *R_i_* values for these Mysticeti are similar, ranging from 0.506 to 0.591 compared to *H. amphibius* and from 0.058 to 0.074 compared to *B. edeni*, apart from *E. glacialis* and *M. novaeangliae*. This suggests that the chromosomal structures of these genomes are conserved.

**Fig. 3.**
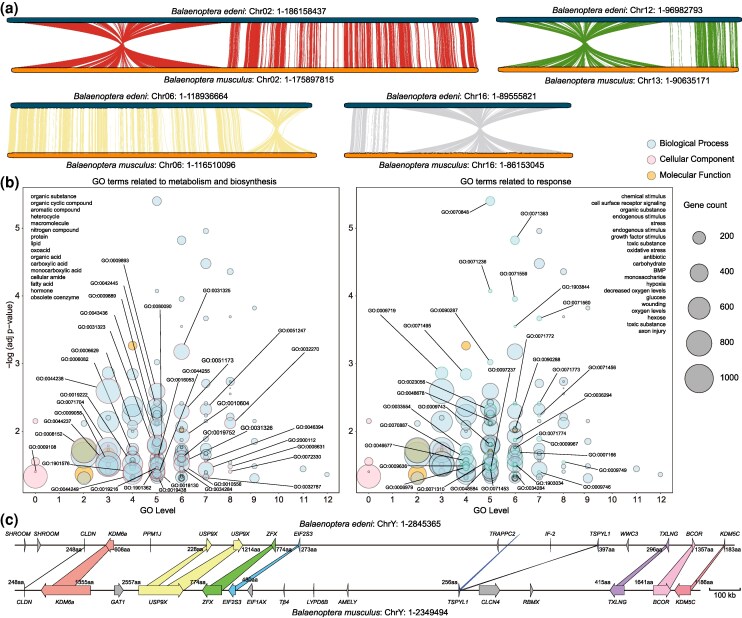
Specific chromosome structures and variations between Eden's and blue whales. a) Inversion macro-fragments in four chromosomes of Eden's whale compared to those of the blue whale. b) Top 30% GO functional enrichment, based on genes within the four inversion macrofragments of Eden's whale, showing their main functions of metabolism, biosynthesis, and responses. c) Gene synteny of the Y chromosomes of Eden's and blue whales.

Our syntenic analysis indicates that the chromosomal structure of *B. edeni* closely resembles that of *B. musculus* (Fig. 2a and b), with four macrofragment inversions identified at one end of four chromosomes (Fig. 3a). Functional enrichment analysis found that the genes located in these inversion blocks (Table S5) are primarily involved in the metabolism and biosynthesis of various substances, as well as responses to substances, stress, and stimuli (Fig. 3b, Table S6). Most of these genes are associated with cellular, primary, and organic compound metabolisms and their regulation. Additional pathways related to macromolecule, nitrogen compound, protein, lipid, acid, and hormone metabolism and biosynthesis were also identified, alongside pathways related to cellular responses to chemicals, organic compounds, carbohydrates, growth factors, toxins, oxygen, and oxidation (Fig. 3b, Table S6). Our comparison of the microsynteny between the male allosomes (ChrY) of *B. edeni* and *B. musculus* (Fig. 3c) revealed their similar length and gene count, and their nine shared genes. Most of the shared genes are arranged in the same direction, with only *TSPYL1* being inverted. Furthermore, we found that most genes of *B. edeni* have shorter lengths and fewer introns while retaining their functional domains (Fig. 3c). For instance, the protein of the *KDM6A* gene contains 608 amino acids (aa) in *B. edeni* and 1,355 aa in *B. musculus*, with the transcript containing nine and 28 introns, respectively. The gene *ZFX* has identical amino acid counts in both genomes but contains 5 and 10 introns in *B. edeni* and *B. musculus*, respectively. The factors affecting these gene sizes and intron differences, as well as the biological implications of these structural changes, remain unknown.

## Discussion

### Macro-Fragment Inversion Drive Speciation and Adaptation

Chromosomal rearrangements—including fusion, fission, duplication, deletion, translocation, and inversion—drive reproductive barriers and speciation. These changes either alter chromosome numbers (through fusion/fission) or modify intra-chromosomal architecture (e.g., duplications and inversions), as demonstrated across multiple systems (Feder and Nosil 2009; De Vos et al. 2020; Lucek et al. 2023). Among these, chromosomal inversions—reversals of gene order within genomic regions—can promote adaptation by suppressing recombination between alternate rearrangements. This preserves adaptive allele combinations and genetic incompatibilities, facilitating speciation (Hoffmann and Rieseberg 2008; Poikela et al. 2024). Specifically, inversions drive divergence through maintaining polymorphisms via balancing selection, shielding co-adapted gene complexes from introgressive gene flow, and accelerating reproductive isolation (Noor et al. 2001; Navarro and Barton 2003; Faria et al. 2019). Despite highly conserved karyotypes across balaenopterid whales, we detected species-specific macro-fragment inversions in every genome analyzed (Fig. 2a). These species-specific inversions between balaenopterids create recombination barriers homologous to those implicated in speciation (Faria et al. 2019). While they likely contributed to balaenopterid divergence, further genomic validation is needed to confirm their causal roles.

Chromosomal rearrangements—including inversions documented in marine mammals—can mediate environmental adaptation by altering gene function (Zhang et al. 2020), primarily through suppressing allelic recombination, disrupting reading frames, and modifying gene expression (Kirkpatrick and Barton 2006; Villoutreix et al. 2021; Wright and Schaeffer 2022). Our functional enrichment analysis of inversion regions between *B. edeni* and *B. musculus* revealed two dominant functional categories: (i) metabolism and biosynthesis pathways (macromolecules, nitrogen compounds, proteins, lipids, acids, and hormones) critical for cetacean growth, development, and body size regulation (Sun et al. 2022); and (ii) stress–response systems involving chemical/organic compounds, carbohydrates, growth factor signaling, toxin detoxification, and oxygen homeostasis (Fig. 3b and c). Both functional clusters show established links to cetacean adaptation, metabolism, and development (Bukhman et al. 2024; Charlanne et al. 2025; Zhang et al. 2025), leading us to propose that these inversions contribute to the body size divergence between *B. edeni* and *B. musculus* (Kato and Perrin 2009; Sears and Perrin 2009). However, the limited tissue types and suboptimal RNA quality of the specimen used in this study highlight the need for future transcriptomic and proteomic studies to validate the functional impacts of these inversions.

### Unexpected Complex Genome Rearrangement of Bryde's Whale

Our syntenic analyses revealed unexpectedly large-scale complex rearrangements in the Bryde's whale genome, characterized by prevalent intra-chromosomal inversions and inter-chromosomal insertions, high rearrangement index values, and minimal conserved blocks (Fig. 2b to d). This substantial syntenic discrepancy with *B. edeni* particularly unexpected given their relatively recent divergence (7.84 Ma). Yuan et al. (2021) assembled the genome of *B. brydei* from Illumina short reads and Hi-C data, but they did not analyze the synteny or report chromosomal rearrangements. We examined the assembly of Bryde's whale and found that their Hi-C contact heatmap is clear (Yuan et al. 2021). The mechanistic basis for these rearrangements could be complex, potentially involving DNA recombination, translocations, or aberrant repair/replication processes (Carvalho and Lupski 2016; Berdan et al. 2024), but unlikely attributable to transposable elements given their comparably low abundance in Bryde's whale versus *B. musculus* and *B. physalus* (Fig. 1f, Fig. S1f) despite known TE-mediated rearrangement mechanisms (Jurka et al. 2011; Ip et al. 2021; Schmitz and Querques 2024). However, given the large-scale rearrangements compared to other balaenopterids and the outgroup *Hippopotamus*, we could not rule out the possibility that the genome was misassembled when the genomic scaffolds were anchored to the Hi-C data. Sequencing an additional Bryde's whale individual is therefore essential to exclude this artifact and confirm the biological reality of these rearrangements.

### Bryde's Whale as a Distinct Species

The taxonomy of the Bryde's whale complex remains contentious. While some morphological studies have suggested that Eden's and Bryde's whales are the same species (Andrews 1918; Junge 1950; Kato and Perrin 2009; Kershaw et al. 2013), previous phylogenetic studies using mitogenomes supported their separation (Wada et al. 2003; Sasaki et al. 2006; Luksenburg et al. 2015; Rosel et al. 2021). However, *B. brydei* has not been formally recognized due to the limited data for genomic comparison (Wada et al. 2003; Rosel et al. 2021). Coupled with earlier morphological and phylogenetic studies, our genomic comparisons provided a compelling case for recognizing Eden's and Bryde's whales as distinct species:

1. The genetic distance of the mitochondrial gene *cox1* between Eden's and Bryde's whales is 2.94%, exceeding the threshold commonly used for species delimitation in mammals (2%) (Hebert et al. 2003; Luo et al. 2011), and substantially larger than that between *B. edeni* and *B. ricei* (1.81%), as well as among *Eubalaena* whales (0.52% to 0.86%).
2. Our phylogenomic analysis, based on phylogenetic analysis using 3,360 single-copy orthogroups, indicated that the phylogenetic distance between *B. edeni* and *B. brydei* is greater than those observed among other closely related mysticete species, although we found that *B. edeni* is sister to *B. brydei*, rather than to *B. ricei* as proposed by prior mitogenomic studies (Wada et al. 2003; Sasaki et al. 2006; Luksenburg et al. 2015; Rosel et al. 2021).
3. Our calibration of the multigene phylogenetic tree using fossil records indicated that the common ancestor of *B. edeni* and *B. brydei* diverged about 7.84 Ma during the late Miocene, which occurred before the divergence of *Phocoena sinus* and *P. phocoena* around 4.47 Ma.
4. Cranial characteristics have been applied to delimit the Bryde's whale complex (Wada et al. 2003; Yamada et al. 2006, 2008; Luksenburg et al. 2015; Rosel et al. 2021). The ascending of the maxilla is slender and round in *B. edeni*, and broadens only slightly posteriorly in *B. ricei*, while it widens to become squarish in *B. brydei* and *B. omurai*. Additionally, the posterior end of the premaxilla is broad and contacts the frontal in *B. edeni*, whereas it is slender and also reaches the frontal in *B. brydei*, but it fails to reach the frontal in *B. omurai*. These morphological features support the recognition that *B. brydei*, *B. edeni*, *B. omurai*, and *B. ricei* are distinct species in the Bryde's whale complex. Therefore, the formal reinstatement of *B. brydei* as a species is warranted.
5. The syntenic analyses revealed large-scale complex rearrangements in *B. brydei* compared to *B. edeni*, which are potentially due to the misassembly of Bryde's whale genome, which needs confirmation.

### Balaenopterid Phylogeny and Evolution of Bryde's Whale Complex

The evolutionary relationships within Balaenopteridae remain debated despite extensive research. Our phylogenomic analysis using single-copy orthogroups corroborates previous genomic studies (Sasaki et al. 2006; Árnason et al. 2018; McGowen et al. 2020), confirming the paraphyletic nature of this family with gray and humpback whales (*E. robustus* and *M. novaeangliae*) nested within *Balaenoptera* lineages. Notably, the gray whale shows an early divergence time of 16.13 Ma preceding most *Balaenoptera* speciation events yet maintains close phylogenetic affinity to the major clade of Balaenopteridae, including Bryde's whale complex, humpback, fin, and blue whales. Árnason et al. (2018) suggested that gray and humpback whales originated within Balaenopteridae, advocating for their classification under *Balaenoptera* based on genomic data, while their unique morphological and ecological traits challenge this view, reflecting broader systematic disagreements. Moreover, the phylogenetic relationships between gray whales and other Balaenopteridae species remain disputed, with different genetic analyses offering conflicting topologies lacking strong support (Hassanin et al. 2012; Árnason et al. 2018; Wolf et al. 2022). Previous studies suggested that these conflicting topologies likely result from evolutionary complexities such as introgression and incomplete lineage sorting (Nikaido et al. 2006; Sasaki et al. 2005; Furni et al. 2024), highlighting the difficulties in resolving relationships between Balaenopteridae and Eschrichtiidae. Therefore, these findings underscore the necessity for integrative genomic and morphological analyses to resolve long-standing controversies in cetacean systematics.

Within the Bryde's whale complex, studies based on mitochondrial control region sequences have yielded conflicting topologies: some suggested *B. brydei* clusters with sei whales (*B. borealis*) as sister to *B. edeni* (Wada et al. 2003; Luksenburg et al. 2015), while others indicated closer *B. brydei–B. edeni* affinity (Sasaki et al. 2006). Recent analyses further complicated this issue with weakly supported groupings (Rosel et al. 2021). These conflicting topologies, derived from limited sequence data, have impeded understanding of evolutionary relationships within the Bryde's whale complex. Our whole-genome phylogeny definitively resolves *B. brydei* and *B. edeni* as sister lineages with robust support, revealing their clear divergence from *B. ricei* and providing crucial insights into this taxonomically challenging group. Additionally, our demographic reconstruction revealed a highly supported divergence history that is consistent with the phylogenomic analyses and no subsequent population bottlenecks (Fig. 1d). This scenario reflects the conserved genomic architecture between *B. ricei* and *B. edeni* (Rosel et al. 2021) but fails to explain the extensive chromosomal rearrangements observed in *B. brydei* relative to its congeners. Furthermore, the unavailability of genomes for *B. omurai* and *B. borealis* necessitates continued investigation into the evolutionary relationships within the Bryde's whale complex. Genomic studies of these two species will advance our understanding of the phylogenetic structure and speciation processes of the Bryde's whale complex, while also providing critical insights into cetacean evolution.

## Conclusion

We assembled a high-quality chromosomal-level genome of *B. edeni*, achieving 99.70% complete and 0.11% fragmented BUSCOs for the assembly. The predicted gene models show 97.50% complete and 0.30% fragmented BUSCOs. Phylogenomic analysis established that *B. edeni* is phylogenetically most closely related to *B. brydei*, forming a clade that is sister to *B. ricei*. Calibration of the tree indicated that the common ancestor of *B. edeni* and *B. brydei* diverged approximately 7.84 Ma during the late Miocene, whereas the split between their ancestral lineage and *B. ricei* occurred earlier, around 10.49 Ma. Demographic reconstruction revealed a scenario indicating that *B. brydei* and *B. edeni* shared a most common ancestor at t1, and this common ancestor is sister to *B. ricei*, which diverged from their most common ancestor at t2. Syntenic analyses revealed that macro-fragment inversions may play a role in the speciation of balaenopterid whales and identified unexpected large-scale complex genome rearrangements in Bryde's whale compared to other balaenopterids, but we cannot rule out the possibility that the genome was misassembled. Functional enrichment analysis of the inversion regions between *B. edeni* and *B. musculus* revealed that the genes located in these inversion blocks are primarily involved in the metabolism and biosynthesis of various substances, as well as responses to substances, stress, and stimuli. Our assembly and analyses provide fundamental genomic resources for further genetic and evolutionary research on cetaceans, offering comprehensive insights into the evolution, speciation, and taxonomy of the family Balaenopteridae and the Bryde's whale complex.

## Materials and Methods

Supplementary material online contains details of materials and methods. In summary, the tissues from an Eden's whale, including blubber, inner muscle, liver, outer muscle, pelvis bone, and pelvis bone fat, were collected from the carcass stranded in Port Shelter, Hong Kong, on 2023 July 31. Genomic DNA was extracted from the inner muscle for Illumina, PacBio HiFi, and Hi-C sequencing, followed by hybrid assembly using integrated bioinformatic pipelines. Total RNA isolated from all tissues was subjected to transcriptomic sequencing to support gene model prediction. The phylogenomic analysis and divergence time estimation were performed using single-copy orthologs of 25 selected cetaceans and hippo as the outgroup. Then, the analyses of divergence scenarios, chromosomal synteny, rearrangement indices, and gene family expansion/contraction were conducted to study balaenopterid taxonomy and speciation.

## Supplementary Material

msaf234_Supplementary_Data

## Data Availability

The Illumina, PacBio HiFi, and RNA sequencing data have been deposited in the National Centre for Biotechnology Information (NCBI) Sequence Read Archive under the BioProject PRJNA1110565. The genome assembly has been deposited at GenBank with accession number JBDJPH000000000. The genome annotation is available in Figshare under DOI: 10.6084/m9.figshare.25801600.
