# Supplementary material for: Chromosome-Level Genome Assembly of Eden's Whale Clarifies the Taxonomy and Speciation of Bryde's Whale Complex
Source: Mol Biol Evol. 2025 Sep 18;42(10):msaf234. doi: 10.1093/molbev/msaf234 (PMC12492004; doi:10.1093/molbev/msaf234)
Supplement: msaf234_Supplementary_Data [file msaf234_supplementary_data.zip › Supplementary_Material_.pdf]

Materials and Methods

Sample Collection

The protocols and procedures used in this study were ethically reviewed and approved by the Research Ethics Committee of Hong Kong Baptist University, Hong Kong SAR, China, under permission number SCI-BIOL-2023-24_008. Tissues from an Eden's whale were collected, including blubber, inner muscle, liver, outer muscle, pelvis bone, and pelvis bone fat. The whale, morphologically identified as a male, was stranded and died in Port Shelter, Hong Kong, on July 31, 2023, and the tissue samples were freshly dissected and stored at Ocean Park Hong Kong and kept at -80 °C.

Genomic and Transcriptomic Sequencing

Genomic DNA was extracted from the muscle tissue using the CTAB method (Stewart & Via, 1993) for Illumina and PacBio HiFi sequencing. DNA quality was assessed using electrophoresis on 1.0% agarose gels and quantified using a NanoDrop ND-1000 spectrophotometer (Thermo Scientific, USA). The DNA sample underwent library construction for Illumina sequencing with an insert size of 350 bp using the Truseq Nano DNA HT Sample Preparation Kit (Illumina USA), following the manufacturer's protocol. The library was sequenced on an Illumina NovaSeq 6000 sequencer (Illumina, USA) to produce 150 bp paired-end reads at Novogene (Tianjin, China). For PacBio HiFi sequencing, the DNA library was constructed using the SMRTbell Express Template Prep Kit 2.0, following the manufacturer's protocol. Sequencing was performed on one SMRT Cell of a PacBio Revio sequencing plate at Novogene (Tianjin, China). For Hi-C sequencing, the muscle tissue was also utilized for library construction with an insert size of 350 bp, following the same protocol as the Illumina sequencing. The library was sequenced on an Illumina NovaSeq 6000 sequencer (Illumina, USA) to produce 150 bp paired-end reads at Novogene (Tianjin, China). The total RNA of each tissue mentioned above was extracted using the Trizol reagent (TAKARA, Japan), with RNA quality and quantity assessed similarly to the DNA samples. Ribosomal RNA was removed from total RNA using the NEBNext Ultra RNA Library Prep Kit for Illumina (NEB, USA), and RNA molecules were

fragmented to 250-300 bp and reverse-transcribed into cDNA. The constructed libraries were paired-end sequenced on the Illumina NovaSeq 6000 to generate 150 bp reads.

Genome Size Prediction

Illumina raw reads (Table S1) were trimmed with Trimmomatic v0.39 (Bolger et al., 2014) (quality score < 30, length < 40 bp). Clean data were used to generate a 21 *k*-mer histogram using Jellyfish v2.2.028 (Marçais & Kingsford, 2011), and GenomeScope v2.0 (Ranallo-Benavidez et al., 2020) was employed to estimate genome size.

Mitogenome Assembly, Annotation, and Genetic Distance

The raw reads of Illumina sequencing (Table S1) were trimmed with Trimmomatic v0.39 (Bolger et al., 2014) (quality score < 30, length < 40 bp). The mitogenome of *B. edeni* was assembled using NOVOPlasty v3.2 (Dierckxsens et al., 2017) with the *B. edeni cox1* sequence (GQ856370) and mitogenome (AB201258) as the seed and reference sequences, respectively. Mitogenome annotation was performed using MITOS2 v2.1.9 (Donath et al., 2019), embedded in the Galaxy online server (https://usegalaxy.eu/). Pairwise genetic distances of *cox1* among the published mitogenomes of cetaceans, including *B. edeni* and *B. brydei*, were estimated using the Kimura-2-parameter (K2P) model implemented in MEGA v7.0 (Kumar et al., 2016).

Draft Genome and Chromosome Assembly

De novo assembly of HiFi reads (Table S1) was performed using HiFiasm v0.18.9 (Cheng et al., 2021) with a coverage of 49×, based on the predicted genome size. Potential alternative heterozygous contigs were eliminated using Purge_Haplotigs v1.1.3 (Roach et al., 2018). Assembly statistics were assessed using QUAST v5.2.0 (Gurevich et al., 2013). The completeness of the final genome assembly was evaluated with Compleasm v0.2.6 (Huang & Li, 2023) against the Cetartiodactyla_odb10 database under default settings.

Raw reads from HiC sequencing (Table S1) were trimmed using Trimmomatic v0.39 (Bolger et al., 2014) (quality score < 30, length < 40 bp). High-quality reads were identified with HiC-Pro v2.10

(Servant et al., 2015), and duplications were removed using the Juicer pipeline v1.5 (Durand et al., 2016a) under default settings. Genomic scaffolding was performed using the 3D *de novo* assembly pipeline (Dudchenko et al., 2017) under the haploid genome model. Pseudo-chromosomal linkage groups were checked, and several corrections were made using Juicebox v1.11.08 (Durand et al., 2016b) to ensure that scaffolds within the same pseudo-chromosomal linkage groups conformed to the Hi-C linkage characteristics. The completeness of the final genome assembly was assessed using Compleasm v0.2.6 (Huang & Li, 2023) against the Cetartiodactyla_odb10 database, and assembly statistics were evaluated with Besides, QUAST v5.2.0 (Gurevich et al., 2013).

Gene Prediction and Functional Annotation

The final genome version was soft masked using RepeatMasker v4.1.2 (http://www.repeatmasker.org/) against repeat libraries from all model organisms in the RepBase v20181026 (Bao et al., 2015) and species-specific repeat libraries in RepeatModeler v2.0.3 (Flynn et al., 2020) under default settings. Genome annotation was performed using MAKER v3.0 (Cantarel et al., 2008). *De novo* and genome-guided transcriptomes of the tissues were assembled to provide transcriptomic evidence. Adapters and low-quality reads (quality score < 20, length < 40 bp) were removed using Trimmomatic v0.39 (Bolger et al., 2014). All clean reads of the six tissues were pooled and used for *de novo* assembly using Trinity v2.8.5 (Grabherr et al., 2011) under default settings. Genome-guided assembly was assembled using Trinity v2.8.5 by aligning RNA sequencing data to the genome using HISAT v2.1.0 (Kim et al., 2015) under default settings. The two transcriptomes were merged using the PASA pipeline v2.2.0 (Haas et al., 2003) following the authors' instructions. The Swiss-Prot release 2024_03 database (https://www.uniprot.org/) combined with selected protein sequences of 25 species in the order Cetartiodactyla, was used as protein evidence (Table S2). Augustus v3.1 (Stanke & Morgenstern, 2005) was used to predict genes in the repeat-masked genome. EVidenceModeler v1.1.1 (Haas et al., 2008) was used to integrate results from different gene predictors. The PASA pipeline v2.2.0 (Haas et al., 2003) was used to enhance EVM gene models by modifying structures and adding UTR annotations with the *de novo* transcriptome. The completeness of predicted gene models was assessed using Compleasm v0.2.6 (Huang & Li, 2023) against the Cetartiodactyla_odb10 database under default settings. Predicted

genes were functionally annotated using EggNOG-MAPPER 5.0 (Huerta-Cepas et al., 2019) under default settings, along with the BLASTP model of Diamond v0.9.24 (Buchfink et al., 2015) targeting the NR database with the E-value of 1e-10.

Phylogenomic Reconstruction and Divergence Time Estimation

To avoid redundant gene homology calls, the longest isoform for each gene was selected to represent that gene. Orthologous groups (OGs) among the genomes of 25 cetacean species and an outgroup, *Hippopotamus amphibius* (Table S2), were inferred using OrthoFinder v2.5.7 (Emms & Kelly, 2019) under default settings. Only single-copy genes with at least 80% taxonomic representation (21 species) in OGs were used to construct the phylogenetic tree. Protein sequences were aligned using MAFFT v7.520 (Katoh & Standley, 2013) with the "auto" strategy under the "normal alignment" mode. Gblocks v0.91b (Talavera & Castresana, 2007) was applied to remove ambiguously aligned fragments, with missing protein or alignment gaps filled with "-". The aligned sequences were concatenated, resulting in an alignment with 27 species with 2,076,590 columns and 269,582 distinct patterns. Maximum-likelihood (ML) analysis was conducted using IQ-TREE v2.1.3 (Nguyen et al., 2015) with the MFP option for model selection and then run for 100,000 ultrafast bootstraps using the best-fit model Q.mammal+F+R10.

Divergence time estimation was conducted based on the ML tree constructed above using MCMCtree implemented in PAML v4.9h (Yang, 2007). Five fossil calibrations were used to constrain the corresponding nodes (Guo et al., 2022): 1) Cetacea-Hippopotamidae split (66 – 52.4 Ma), constrained by the oldest crown artiodactyl *Himalayacetus subathuensis* (Benton et al., 2015); 2) Baleen-toothed whale divergence (36.4 ± 1.5 Ma), anchored to the earliest cetacean fossil record (Lambert et al., 2017); 3) Phocoenidae-Monodontidae separation (19.50 – 7.50 Ma), calibrated using the oldest crown phocoenid *Salumiphocoena stocktoni* (Barnes, 1985) and earliest delphinid *Kentriodon pernix* (Kellogg, 1927); 4) Delphinidae origin (19.50 – 8.50 Ma), defined by *Eodelphinus kabatensis* (Murakami et al., 2014) and the oldest delphinid record of *Kentriodon pernix* (Kellogg, 1927); 5) Mysticeti emergence (34.6 – 33.9 Ma), based on the fossil records of *Toipahautea Waitaki* (Tsai & Fordyce, 2018) and *Llanocetus denticrenatus* (Hospitaleche & Reguero, 2010). The LG model was employed for each partition, with burn-in, sample frequency, and sample

sizes set at 1 million, 1000, and 10,000, respectively, running MCMC for 10 million generations.

Species Divergence Scenarios of Bryde's whale complex

To study the speciation of the Bryde's whale complex, we identified the genome-wide SNPs of *B. edeni*, *B. brydei*, and *B. ricei* with *B. musculus* as reference. To save computer costs, ART-Illumina (Huang et al., 2012) was used to generate the simulated Illumina reads from the complete genome of three species of Bryde's whale complex with a coverage of 2×. The reads were mapped to the most closely related outgroup, the reference genome of *B. musculus* (Table S2), using BWA-MEM2 (Vasimuddin et al., 2019) under default settings, and then the duplicates were removed using Picard v2.27.5 (https://broadinstitute.github.io/picard/). GATK4 (Van der Auwera & O'Connor, 2020) was used for variant calling and SNP filtering, while BCFtools v1.21 (Li, 2011) and the script vcfrandomsample of VCFlib (Garrison et al., 2022) were coupled to subsample SNPs with a parameter r of 0.0052 to randomly select 10,295 SNPs without missing data. Then the final SNP dataset was transformed using the script vcf2DIYABC.py (https://github.com/loire/vcf2DIYABC.py). The matrix was used for the reconstruction of demographic history and biographical pathways leading to the differentiation among lineages using an Approximate Bayesian Computation (ABC) approach (Sunnåker et al., 2013) implemented in DIYABC v2.1.0 (Cornuet et al., 2014) with 10,295 SNPs for simulation differentiation scenarios. Based on phylogenetic topology reconstructed from SNP data and considerations of biological realism, six putative demographic scenarios and null hypotheses (Fig. S3) were established to infer how the Bryde's whale complex had formed and the possible factors that distinguished the scenarios from the tree topology. A total of 6,000,000 simulated datasets were grouped according to each scenario. The probability of each scenario was measured by performing a weighted logistic regression of each scenario's probability to compare the deviations between simulated and observed summary statistics to find the most likely scenario.

Synteny and Rearrangement Index

To investigate the karyotype and chromosomal structure of Balaenopteridae species, microsynteny analysis was conducted among *B. edeni*, *B. brydei*, *B. ricei*, *B. musculus*, *B. acutorostrata*,

Megaptera novaeangliae, *Eschrichtius robustus*, *E. glacialis*, and outgroup *H. amphibius* using MCScanX v1.0.0 (Wang et al., 2012) implemented in the PanSyn pipeline (Yu et al., 2024) with parameters: -e 1e-10, -mk 100, -me 1e-10, -s 2, and visualized using NGenomeSyn v1.41 (He et al., 2023). Besides, macrosynteny comparisons among *B. edeni*, *B. brydei*, and *B. musculus* were made using WGDI v0.74 (Sun et al., 2022) with parameters: E-value = 1e-10, multiple = 1, repeat number = 1, position = end, based on protein pair-wise alignment using Diamond v0.9.24 (Buchfink et al., 2015) with an E-value of 1e-10.

A rearrangement index (R_i) was calculated to quantify the extent of the chromosomal rearrangement relative to the reference genomes, *B. edeni* and *H. amphibius*, respectively, in balaenopterids (Lewin et al., 2024). The calculation was performed based on the collinear results generated from MCScanX. For each chromosome of the target genome, the rearrangement index was calculated as: $R_{CHR} = 1 - (S_{CHR} \times C_{CHR})$, in which S_{CHR} (splitting parameter) represents the maximum proportion of genes from a chromosome located on a single chromosome, and C_{CHR} (combining parameter) refers to the proportion of genes on that chromosome belonging to it. Subsequently, the R_i for each genome is given by the equation: $R_i = \Sigma(R_{CHR})/N$, where R_i represents the rearrangement index for the target genome, and N is the total number of chromosomes in the reference genome. Additionally, the chromosomal splitting index (S_i) and combining index (C_i) for each target genome were calculated as $S_i = \Sigma(1\text{-}S_{CHR})/N$ and $C_i = \Sigma(1\text{-}C_{CHR})/N$, respectively. A higher R_i indicates a greater level of chromosomal rearrangement, and higher S_i and C_i suggest the genome is dominated by interchromosomal and intrachromosomal rearrangements, respectively.

Gene Family Expansion and Contraction

The CAFE5 v1.1 pipeline (Mendes et al., 2020) was used to identify gene family expansions and contractions based on the orthologous groups (OGs) determined by OrthoFinder v2.5.7 (Emms & Kelly, 2019). Gene families generated for each species were filtered to remove families with more than 100 genes in any species, adhering to pipeline requirements. The filtered table and the ultrametric tree of 31 species generated from the phylogenomic analysis served as inputs for CAFE5, with parameters set to “-p -k 7”. Significantly expanded and contracted gene families with a p-value less than 0.05 were selected. GO functional enrichment analysis of these gene families was

performed using TBtools v2.112 (Chen et al., 2023) against the embedded go-basic.obo database v1.2 (https://geneontology.org/docs/download-ontology/), considering a false discovery rate (FDR) < 0.05 as significantly enriched. Visualization was conducted using the R package GOplot v1.0.2 (Walter et al., 2015).

Supplementary Results

Assembly Statistics

The estimated genome size was 2.2 Gb, with a heterogeneity of 0.20% and a duplication rate of 1.26% (Fig. S1A). Assembly of PacBio HiFi sequences yielded a draft genome consisting of 163 contigs with a total length of 2.6 Gb and a GC content of 41.3%. The N50 of the draft genome is 60.8 Mb, with a maximum length of 154.1 Mb and an average length of 16.0 Mb (Table 1). Among the draft assembly, 2.4 Gb (93.60%) was anchored to the chromosomal level based on the Hi-C data, resulting in a final assembly comprising 23 chromosomes (21 autosomes and the allosomes X and Y, 2n = 44) (Fig. 1A & S1B, Table 1 & S2-S3). The lengths of chromosomes range from 2.84 Mb (ChrY) to 188.9 Mb (Chr01) (Table S3). Although the assembly size slightly exceeds the prediction (Fig. S1A), it closely aligns with the genome sizes of the congeneric species, which range from 2.2 to 2.7 Gb with the same chromosome number (Table S2). Mapping of PacBio HiFi reads to the final genome demonstrated high sequencing coverage (43.7×) and an exceptional mapping rate of 99.98% (Table 1). Benchmarking Universal Single-Copy Orthologs (BUSCO) assessment confirmed the high quality of the assembly with 99.70% complete, 98.10% single-copy, 1.60% duplicated, 0.11% fragmented, and 0.19% missing BUSCOs (Table 1). Repetitive elements constituted 46.93% of the assembly, with transposable element (TE) content in *B. edeni* comparable to that of *B. ricei* and *B. acutorostrata*, but significantly higher than in other balaenopterid whales (Fig. S1C). The elevated TE levels in the genomes of *B. edeni*, *B. ricei*, and *B. acutorostrata*, particularly the long interspersed nuclear elements (LINEs), may be a key contributor to their large genome sizes (Fig. S1C). Gene model prediction identified 19,476 protein-coding genes (PCGs) with an average length of 1,618 bp (Table 1). The total gene count across the chromosomes varies, with Chr01 hosting the highest number at 1,887 genes, while ChrY contains only 16 genes (Table S3). Among the PCGs, 19,155 (98.35%) were functionally annotated at least against a public database (Fig. S1D, Table 1 & S3). BUSCO assessment indicated that the gene models represent 97.50% complete, 96.40% single-copy,

1.10% duplicated, 0.30% fragmented, and 2.20% missing BUSCOs (Table 1).

Biological Processes Related to Gene Expansion and Contraction

Gene family expansion and contraction are crucial for understanding the evolution and adaptation of organisms. In the genome of Eden's whale, only 31 gene families were significantly expanded, while 131 families were contracted (Fig. 2, Table S7). The number of expanded families in *B. edeni* is considerably lower than that of other congeneric species, which range from 107 to 230, with most inversions occurring through tandem duplication. Although the functions of most expanded families are diverse and cannot be attributed to specific biological processes, some are associated with transcriptional regulation and the immune system (Table S8). In contrast, the contracted gene families in *B. edeni* cluster into six biological categories, including transcriptional regulation, ribosomal proteins involved in protein synthesis, mitochondrial respiration chain, immune system, growth and development, and olfactory receptors (Fig. 5A, Table S8). The genome of Eden's whale exhibits low copy numbers of ribosomal genes, with ten specific missing gene families, including *RPL22*, *RPL27*, *RPL30*, *RPL32*, *RPL36*, *RPL36A*, *RPL39*, *RPS20*, *RPS24*, and *RPS29* (Table S8). Similarly, certain olfactory receptors, including OR10A5, OR10P1, OR10R2, and OR52B6, are absent in *B. edeni*. Furthermore, contracted families related to growth and development in *B. edeni*, such as ARF4, CXCL1, HINT1, and IFI27L2, maintain high copy numbers in *B. acutorostrata* and *B. bonaerensis*, both of which have small body sizes (Table S8). This suggests that the contraction of these gene families (Fig. 5A, Table S8) in *B. edeni* is not correlated with the body size among balaenopterids. Additionally, we found that most olfactory receptors and immune-related genes also exhibit low copy numbers in the Antarctic minke whale *B. bonaerensis* and fin whale *B. physalus* (Fig. 5A). In contrast, the genome of Bryde's whale contains more expanded (107) and fewer contracted (18) gene families (Fig. 2, Table S7). Notably, among the expanded families of *B. brydei*, 31 are shared with the contracted families of *B. edeni*, primarily related to ribosomal protein and olfactory receptors. GO functional enrichment analysis of the expanded families of *B. brydei* indicated that these gene families are associated with intracellular structures, organic and nitrogen compound metabolisms, and macromolecule metabolic processes (Table S9).

References

Bao, W., Kojima, K. K., & Kohany, O. (2015). Repbase Update, a database of repetitive elements in eukaryotic genomes. *Mobile DNA*, *6*(1), 11.

Barnes, L. G. (1985). Evolution, taxonomy and antitropical distributions of the porpoises (Phocoenidae, Mammalia). *Marine Mammal Science*, *1*(2), 149–165.

Benton, M. J., Donoghue, P. C., Asher, R. J., Friedman, M., Near, T. J., & Vinther, J. (2015). Constraints on the timescale of animal evolutionary history. *Palaeontologia Electronica*, *18*(1), 1–106.

Bolger, A. M., Lohse, M., & Usadel, B. (2014). Trimmomatic: A flexible trimmer for Illumina sequence data. *Bioinformatics*, *30*(15), 2114–2120.

Buchfink, B., Xie, C., & Huson, D. H. (2015). Fast and sensitive protein alignment using DIAMOND. *Nature Methods*, *12*(1), 1.

Cantarel, B. L., Korf, I., Robb, S. M., Parra, G., Ross, E., Moore, B., Holt, C., Alvarado, A. S., & Yandell, M. (2008). MAKER: An easy-to-use annotation pipeline designed for emerging model organism genomes. *Genome Research*, *18*(1), 188–196.

Chen, C., Wu, Y., Li, J., Wang, X., Zeng, Z., Xu, J., Liu, Y., Feng, J., Chen, H., & He, Y. (2023). TBtools-II: A" One for All, All for One" bioinformatics platform for biological big-data mining. *Molecular Plant*. *16*(11), 1733–1742.

Cheng, H., Concepcion, G. T., Feng, X., Zhang, H., & Li, H. (2021). Haplotype-resolved de novo assembly using phased assembly graphs with Hifiasm. *Nature Methods*, *18*(2), 170–175.

Cornuet, J. M., Pudlo, P., Veyssier, J., Dehne-Garcia, A., Gautier, M., Leblois, R., Marin, J-M., & Estoup, A. (2014). DIYABC v2. 0: A software to make approximate Bayesian computation inferences about population history using single nucleotide polymorphism, DNA sequence and microsatellite data. *Bioinformatics*, *30*(8), 1187–1189.

Dierckxsens, N., Mardulyn, P., & Smits, G. (2017). NOVOPlasty: *De novo* assembly of organelle genomes from whole genome data. *Nucleic Acids Research*, *45*(4), e18–e18.

Donath, A., Jühling, F., Al-Arab, M., Bernhart, S. H., Reinhardt, F., Stadler, P. F., Middendorf, M., &

Bernt, M. (2019). Improved annotation of protein-coding genes boundaries in metazoan mitochondrial genomes. *Nucleic Acids Research*, *47*(20), 10543–10552.

Dudchenko, O., Batra, S. S., Omer, A. D., Nyquist, S. K., Hoeger, M., Durand, N. C., Shamim, M. S., Machol, I., Lander, E. S., Aiden, A. P., & Aiden, E. L. (2017). *De novo* assembly of the *Aedes aegypti* genome using Hi-C yields chromosome-length scaffolds. *Science*, *356*(6333), 92–95.

Durand, N. C., Robinson, J. T., Shamim, M. S., Machol, I., Mesirov, J. P., Lander, E. S., & Aiden, E. L. (2016). Juicebox provides a visualization system for Hi-C contact maps with unlimited zoom. *Cell Systems*, *3*(1), 99–101.

Durand, N. C., Shamim, M. S., Machol, I., Rao, S. S., Huntley, M. H., Lander, E. S., & Aiden, E. L. (2016). Juicer provides a one-click system for analyzing loop-resolution Hi-C experiments. *Cell Systems*, *3*(1), 95–98.

Emms, D. M., & Kelly, S. (2019). OrthoFinder: Phylogenetic orthology inference for comparative genomics. *Genome Biology*, *20*(1), 238.

Flynn, J. M., Hubley, R., Goubert, C., Rosen, J., Clark, A. G., Feschotte, C., & Smit, A. F. (2020). RepeatModeler2 for automated genomic discovery of transposable element families. *Proceedings of the National Academy of Sciences*, *117*(17), 9451–9457.

Grabherr, M. G., Haas, B. J., Yassour, M., Levin, J. Z., Thompson, D. A., Amit, I., Adiconis, X., Fan, L., Raychowdhury, R., & Zeng, Q. (2011). Full-length transcriptome assembly from RNA-Seq data without a reference genome. *Nature Biotechnology*, *29*(7), 644–652.

Guo, W., Sun, D., Cao, Y., Xiao, L., Huang, X., Ren, W., Xu, S., & Yang, G. (2022). Extensive Interspecific Gene Flow Shaped Complex Evolutionary History and Underestimated Species Diversity in Rapidly Radiated Dolphins. *Journal of Mammalian Evolution*, *29*(2), 353–367.

Gurevich, A., Saveliev, V., Vyahhi, N., & Tesler, G. (2013). QUAST: Quality assessment tool for genome assemblies. *Bioinformatics*, *29*(8), 1072–1075.

Haas, B. J., Delcher, A. L., Mount, S. M., Wortman, J. R., Smith Jr, R. K., Hannick, L. I., Maiti, R., Ronning, C. M., Rusch, D. B., & Town, C. D. (2003). Improving the Arabidopsis genome annotation using maximal transcript alignment assemblies. *Nucleic Acids Research*, *31*(19),

5654–5666.

Haas, B. J., Salzberg, S. L., Zhu, W., Pertea, M., Allen, J. E., Orvis, J., White, O., Buell, C. R., & Wortman, J. R. (2008). Automated eukaryotic gene structure annotation using EVidenceModeler and the Program to Assemble Spliced Alignments. *Genome Biology*, *9*(1), R7.

He, W., Yang, J., Jing, Y., Xu, L., Yu, K., & Fang, X. (2023). NGenomeSyn: An easy-to-use and flexible tool for publication-ready visualization of syntenic relationships across multiple genomes. *Bioinformatics*, *39*(3), btad121.

Hospitaleche, C. A., & Reguero, M. (2010). First articulated skeleton of *Palaeeudyptes gunnari* from the late Eocene of Isla Marambio (Seymour Island), Antarctica. *Antarctic Science*, *22*(3), 289–298.

Huang, N., & Li, H. (2023). Compleasm: A faster and more accurate reimplementation of BUSCO. *Bioinformatics*, *39*(10), btad595.

Huang, W., Li, L., Myers, J. R., & Marth, G. T. (2012). ART: A next-generation sequencing read simulator. *Bioinformatics*, *28*(4), 593–594.

Huerta-Cepas, J., Szklarczyk, D., Heller, D., Hernández-Plaza, A., Forslund, S. K., Cook, H., Mende, D. R., Letunic, I., Rattei, T., & Jensen, L. J. (2019). eggNOG 5.0: A hierarchical, functionally and phylogenetically annotated orthology resource based on 5090 organisms and 2502 viruses. *Nucleic Acids Research*, *47*(D1), D309–D314.

Katoh, K., & Standley, D. M. (2013). MAFFT multiple sequence alignment software version 7: Improvements in performance and usability. *Molecular Biology and Evolution*, *30*(4), 772–780.

Kellogg, R. (1927). *Kentriodon Pernix: A Miocene Porpoise from Maryland.* US Government Printing Office.

Kim, D., Langmead, B., & Salzberg, S. L. (2015). HISAT: A fast spliced aligner with low memory requirements. *Nature Methods*, *12*(4), 357–360.

Kumar, S., Stecher, G., & Tamura, K. (2016). MEGA7: Molecular evolutionary genetics analysis

version 7.0 for bigger datasets. *Molecular Biology and Evolution*, *33*(7), 1870–1874.

Lambert, O., Martínez-Cáceres, M., Bianucci, G., Di Celma, C., Salas-Gismondi, R., Steurbaut, E., Urbina, M., & De Muizon, C. (2017). Earliest mysticete from the Late Eocene of Peru sheds new light on the origin of baleen whales. *Current Biology*, *27*(10), 1535–1541.

Lewin, T. D., Liao, I. J. Y., & Luo, Y. J. (2024). Annelid comparative genomics and the evolution of massive lineage-specific genome rearrangement in bilaterians. *Molecular Biology and Evolution*, *41*(9), msae172.

Li, H. (2011). A statistical framework for SNP calling, mutation discovery, association mapping and population genetical parameter estimation from sequencing data. *Bioinformatics*, *27*(21), 2987–2993.

Marçais, G., & Kingsford, C. (2011). A fast, lock-free approach for efficient parallel counting of occurrences of *k-mers*. *Bioinformatics*, *27*(6), 764–770.

Mendes, F. K., Vanderpool, D., Fulton, B., & Hahn, M. W. (2020). CAFE 5 models variation in evolutionary rates among gene families. *Bioinformatics*, *36*(22–23), 5516–5518.

Murakami, M., Shimada, C., Hikida, Y., Soeda, Y., & Hirano, H. (2014). *Eodelphis kabatensis* , a new name for the oldest true dolphin *Stenella kabatensis* Horikawa, 1977 (Cetacea, Odontoceti, Delphinidae), from the upper Miocene of Japan, and the phylogeny and paleobiogeography of Delphinoidea. *Journal of Vertebrate Paleontology*, *34*(3), 491–511.

Nguyen, L.-T., Schmidt, H. A., von Haeseler, A., & Minh, B. Q. (2015). IQ-TREE: A Fast and Effective Stochastic Algorithm for Estimating Maximum-Likelihood Phylogenies. *Molecular Biology and Evolution*, *32*(1), 268–274.

Ranallo-Benavidez, T. R., Jaron, K. S., & Schatz, M. C. (2020). GenomeScope 2.0 and Smudgeplot for reference-free profiling of polyploid genomes. *Nature Communications*, *11*(1), 1432.

Roach, M. J., Schmidt, S. A., & Borneman, A. R. (2018). Purge Haplotigs: Allelic contig reassignment for third-gen diploid genome assemblies. *BMC Bioinformatics*, *19*(1), 460.

Stanke, M., & Morgenstern, B. (2005). AUGUSTUS: A web server for gene prediction in eukaryotes that allows user-defined constraints. *Nucleic Acids Research*, *33*(suppl_2), W465–W467.

Stewart, C. N., & Via, L. E. (1993). A rapid CTAB DNA isolation technique useful for RAPD fingerprinting and other PCR applications. *Biotechniques*, *14*(5), 748–751.

Sun, P., Jiao, B., Yang, Y., Shan, L., Li, T., Li, X., Xi, Z., Wang, X., & Liu, J. (2022). WGDI: A user-friendly toolkit for evolutionary analyses of whole-genome duplications and ancestral karyotypes. *Molecular Plant*, *15*(12), 1841–1851.

Sunnåker, M., Busetto, A. G., Numminen, E., Corander, J., Foll, M., & Dessimoz, C. (2013). Approximate Bayesian computation. *PLoS Computational Biology*, *9*(1), e1002803.

Talavera, G., & Castresana, J. (2007). Improvement of phylogenies after removing divergent and ambiguously aligned blocks from protein sequence alignments. *Systematic Biology*, *56*(4), 564–577.

Tsai, C.-H., & Fordyce, R. E. (2018). A new archaic baleen whale *Toipahautea waitaki* (early Late Oligocene, New Zealand) and the origins of crown Mysticeti. *Royal Society Open Science*, *5*(4), 172453.

Van der Auwera, G. A., & O'Connor, B. D. (2020). *Genomics in the cloud: using Docker, GATK, and WDL in Terra*. O'Reilly Media, USA.

Vasimuddin, M., Misra, S., Li, H., & Aluru, S. (2019). Efficient architecture-aware acceleration of BWA-MEM for multicore systems. *IEEE Parallel and Distributed Processing Symposium (IPDPS)*, 314–324

Walter, W., Sánchez-Cabo, F., & Ricote, M. (2015). GOplot: An R package for visually combining expression data with functional analysis. *Bioinformatics*, *31*(17), 2912–2914.

Wang, Y., Tang, H., DeBarry, J. D., Tan, X., Li, J., Wang, X., Lee, T., Jin, H., Marler, B., & Guo, H. (2012). MCScanX: A toolkit for detection and evolutionary analysis of gene synteny and collinearity. *Nucleic Acids Research*, *40*(7), e49–e49.

Yang, Z. (2007). PAML 4: Phylogenetic analysis by maximum likelihood. *Molecular Biology and Evolution*, *24*(8), 1586–1591.

Yu, H., Li, Y., Han, W., Bao, L., Liu, F., Ma, Y., Pu, Z., Zeng, Q., Zhang, L., & Bao, Z. (2024). Pan-evolutionary and regulatory genome architecture delineated by an integrated macro-and

microsynteny approach. *Nature Protocols*, *19*(6), 1–56.

Supplementary Figures

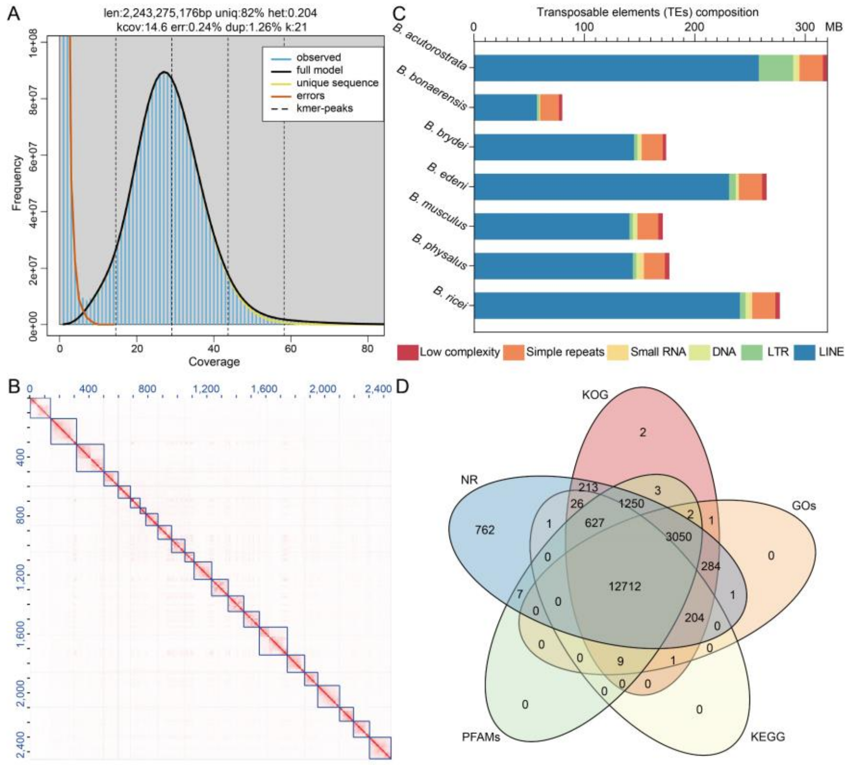

Fig. S1 Genome size prediction, structure, and functional annotation of Eden's whale. (A) Predicted genome size and heterogeneity. (B) A Hi-C interaction heatmap showing the 22 inferred chromosomes (excluding chromosome Y, which is too small to display). (C) Composition of transposable elements (TEs) in *Balaenoptera* species. (D) Venn diagram showing the functional annotation of the predicted gene models.

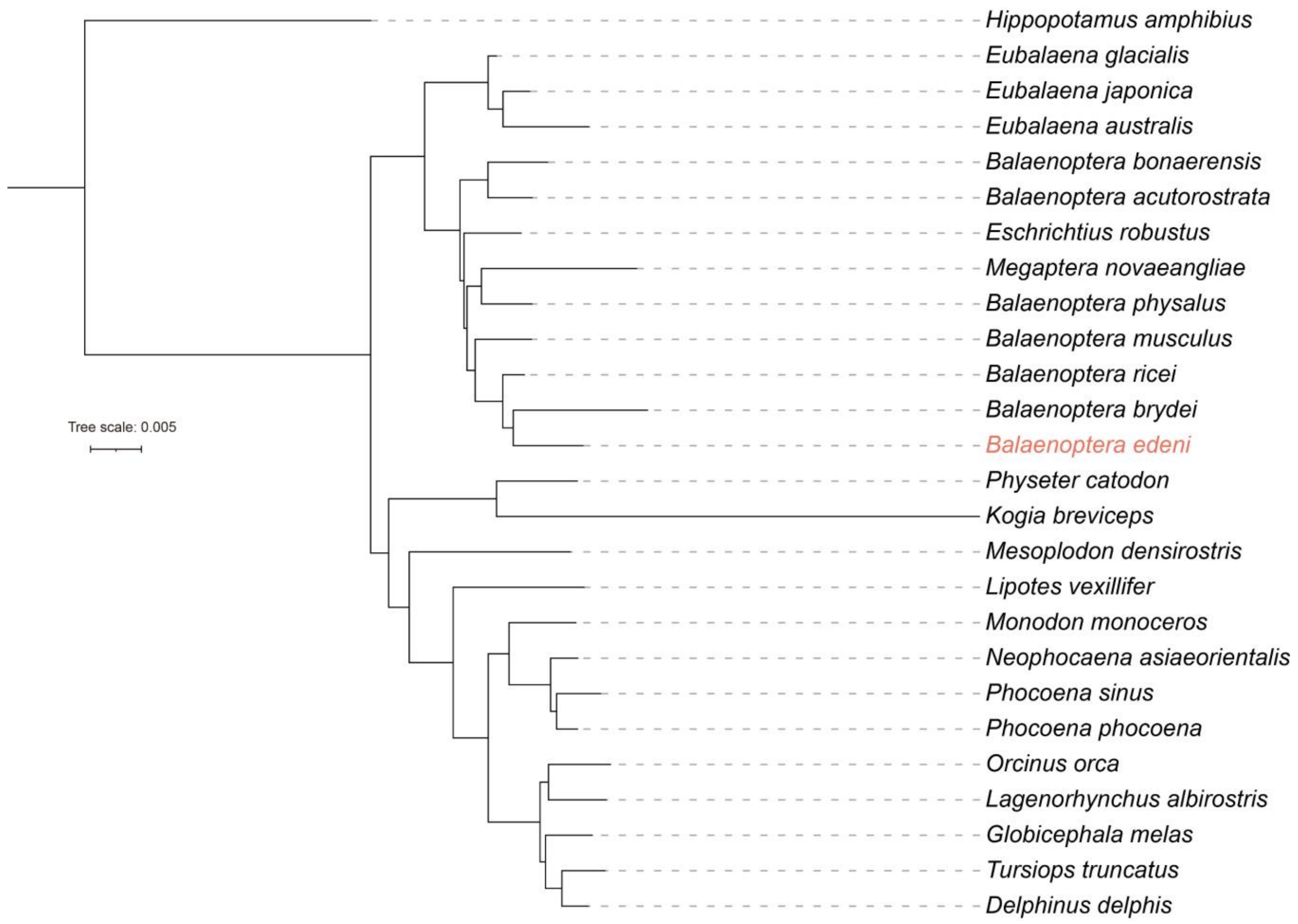

Fig. S2 Phylogenomic relationships of Eden's whale. The tree was constructed using 3,360 single-copy orthogroups based on the maximum-likelihood analysis with the MFP option for model selection and then run for 100,000 ultrafast bootstraps using the best-fit model Q.mammal+F+R10. The bootstrap values are 100 at all nodes. Sources and characteristics of the genomes are included in Table S2.

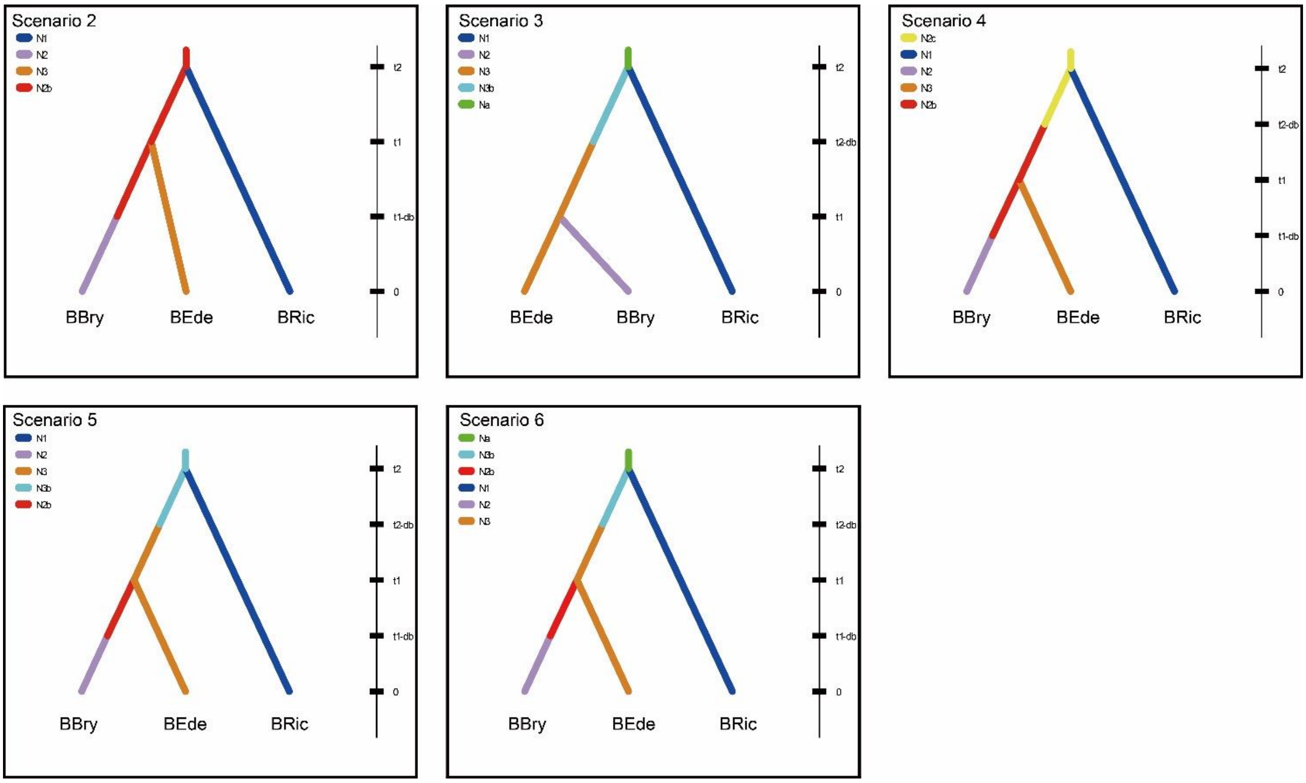

Fig. S3 Putative divergence scenarios 2 to 6 based on the SNP dataset (N=10,295). N1, *Balaenoptera ricei* (BRic); N2, *B. brydei* (BBry); N3, *B. edeni* (BEde); Na, common ancestor; N2b, N2c, and N3b, putative mimics demographic bottlenecks; t2, time point of first divergence event from the ancestor; from t2 to 0, from the ancient to present.

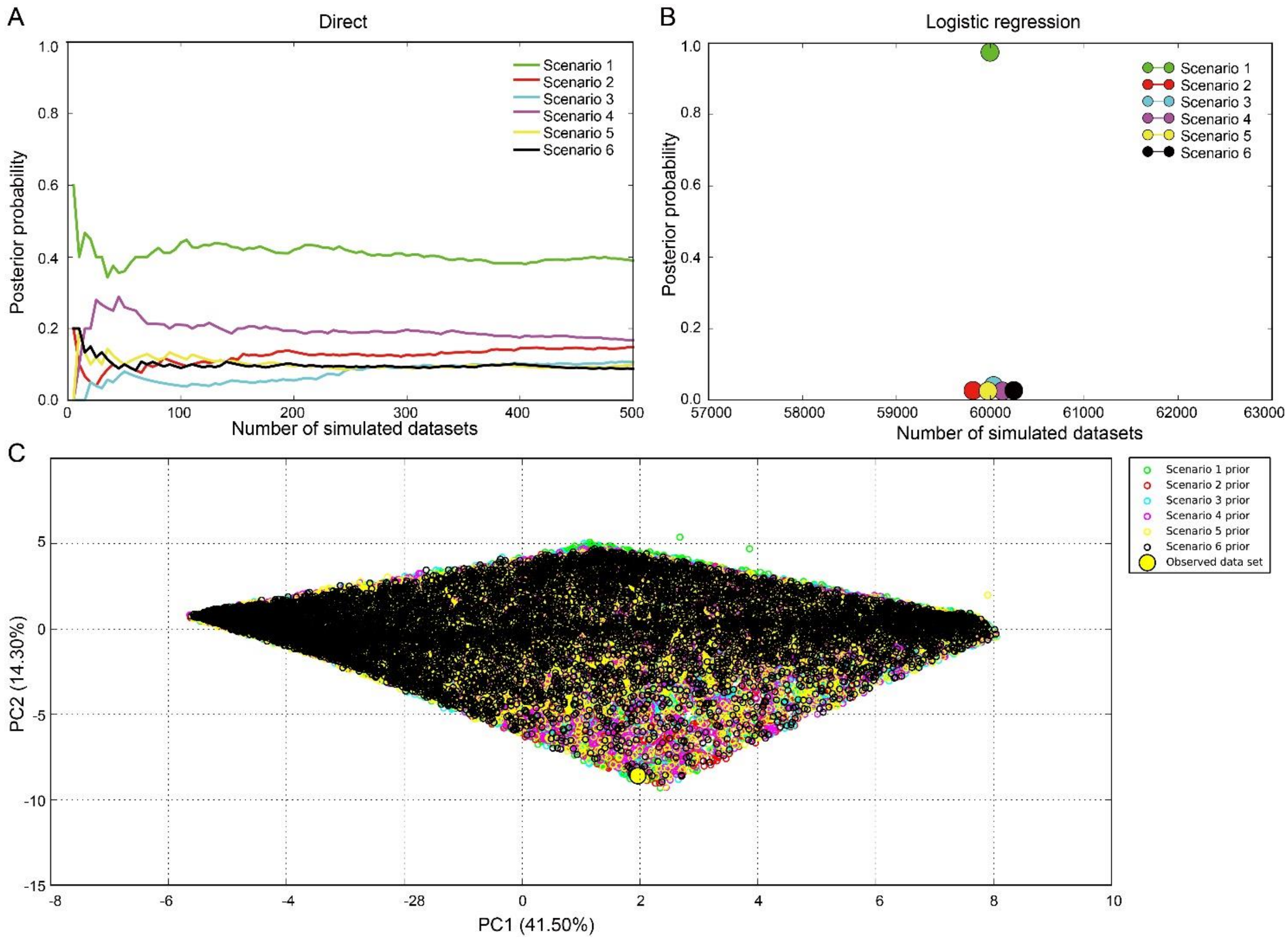

Fig. S4 Posterior probability and principal components analysis of the putative divergence scenarios 1 to 6 based on the sampled SNP dataset (N=10,295). (A-B) Posterior probability of the putative divergence scenarios. (C) Principal components analysis of all putative divergence scenarios.

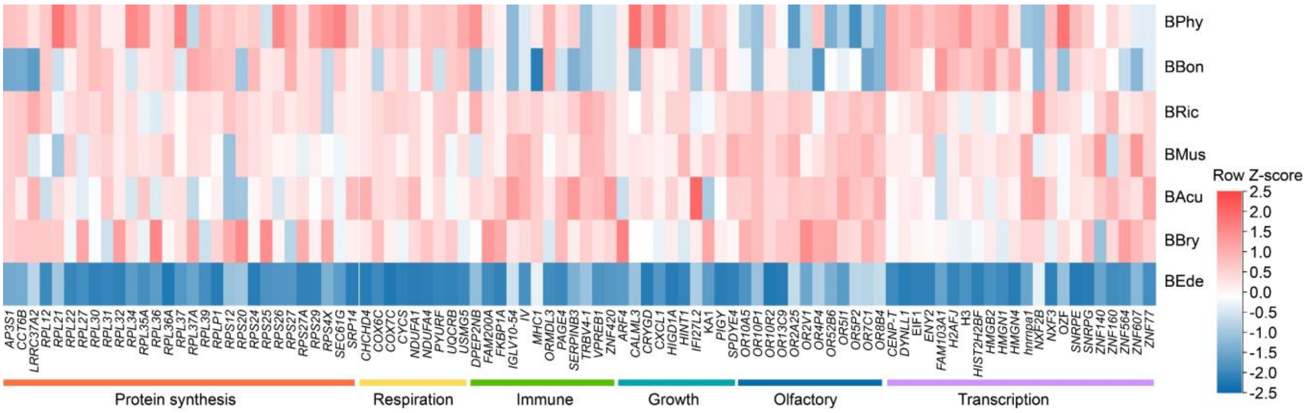

Fig. S5 Contracted gene families in Eden's whale compared to congeneric species with a larger body size. The heat map of the contracted gene families and their related functions. Abbreviation: BAcu, *B. acutorostrata*; BBon, *B. bonaerensis*; BBry, *B. brydei*; BEde, *B. edeni*; BMus, *B. musculus*; BPhy, *B. physalus*; Bric, *B. ricei*.
